# Quantitative analysis of MBW complex formation in the context of trichome patterning

**DOI:** 10.3389/fpls.2024.1331156

**Published:** 2024-03-05

**Authors:** Bipei Zhang, Anna Deneer, Christian Fleck, Martin Hülskamp

**Affiliations:** ^1^ Key Laboratory of Tropical and Subtropical Flowers and Landscape Plants of Guangdong Higher Education Institutions/College of Horticulture and Landscape Architecture, ZhongKai University of Agriculture and Engineering, Guangzhou, China; ^2^ Biometris, Department of Mathematical and Statistical Methods, Wageningen University, Wageningen, Netherlands; ^3^ Spatial Systems Biology Group, Center for Data Analysis and Modeling, University of Freiburg, Freiburg, Germany; ^4^ Botanical Institute, Biocenter, Cologne University, Cologne, Germany

**Keywords:** *Arabidopsis thaliana*, trichome patterning, protein interactions, dissociation constant, protein complex formation, mathematical modelling

## Abstract

Trichome patterning in *Arabidopsis* is regulated by *R2R3MYB*, *bHLH* and *WDR* (MBW) genes. These are considered to form a trimeric MBW protein complex that promotes trichome formation. The MBW proteins are engaged in a regulatory network to select trichome cells among epidermal cells through R3MYB proteins that can move between cells and repress the MBW complex by competitive binding with the R2R3MYB to the bHLHL protein. We use quantitative pull-down assays to determine the relative dissociation constants for the protein-protein interactions of the involved genes. We find similar binding strength between the trichome promoting genes and weaker binding of the R3MYB inhibitors. We used the dissociation constants to calculate the relative percentage of all possible complex combinations and found surprisingly low fractions of those complexes that are typically considered to be relevant for the regulation events. Finally, we predict an increased robustness in patterning as a consequence of higher ordered complexes mediated by GL3 dimerization.

## 1 Introduction

The MBW complex consisting of MYB and bHLH transcription factors associated with a WD40 repeat protein drives multiple traits in a range of plant species, among which metabolic pathways and cell differentiation [Ramsay and Glover (2005); Robinson and Roeder (2015); Xu et al. (2015); Zhang et al. (2019); Zhao et al. (2008); Koornneef (1981)]. Mutation and duplication events in the genes encoding the MBW proteins have given rise to a wide array of developmental regulatory mechanisms that provide the flexibility needed to generate the different epidermal cell types found in plants [Feller et al. (2011); Robinson and Roeder (2015); Ramsay and Glover (2005); Serna and Martin (2006)]. In *Arabidopsis thaliana* the MBW complex is involved in anthocyanin biosynthesis, seed coat mucilage, seed coat pigmentation, and trichome and root hair patterning [Ramsay and Glover (2005); Zhang et al. (2019)].

An excellent system in which to study epidermal cell differentiation and patterning is that of trichome formation [Pesch and Hülskamp (2009); Hülskamp (2004); Pattanaik et al. (2014)]. Trichomes patterning takes place early during leaf development. Among apparently equivalent epidermal cells, trichomes emerge close but not immediately next to each other [Hülskamp et al. (1994)]. Trichome patterning is regulated by a regulatory network of genes that either promote or suppress trichome formation. The WD40 protein TRANSPARENT TESTA GLABRA1 (TTG1) [Walker et al. (1999); Galway et al. (1994); Koornneef (1981)], the R2R3MYB GLABRA1 (GL1) [Oppenheimer et al. (1991)], and the basic helix-loop-helix (bHLH)-like transcription factor GLABRA3 (GL3) act as the major positive regulators [Hülskamp et al. (1994); Koornneeff et al. (1982); Payne et al. (2000)]. Negative regulators of trichomes are encoded by small R3MYBs, in particular TRIPTYCHON (TRY) and CAPRICE (CPC) [Hülskamp et al. (1994); Schellmann et al. (2002); Wada et al. (1997); Gan et al. (2011); Kirik et al. (2004a); Kirik et al. (2004b); Schellmann et al. (2002); Tominaga et al. (2008); Wang et al. (2007); Wang et al. (2008); Wang et al. (2010); Wester et al. (2009)]. This mechanism by which these genes can create a *de novo* pattern has been explored in various theoretical models [Benitez et al., 2007, 2008); Bouyer et al. (2008); Digiuni et al. (2008); Okamoto et al. (2020); Balkunde et al. (2020); Chopra et al. (2019)]. These are based on an activator-inhibitor (AI model), an activator depletion model (AD model) [Pesch and Hülskamp (2009)], or a combination of both [Balkunde et al. (2020)]. The proteins involved show complex protein interaction patterns. The WD40 protein TTG1 and the R2R3MYB protein GL1 bind to the bHLH protein GL3 [Pesch et al. (2015)]. This protein complex is considered to promote trichome formation [Morohashi et al. (2007); Morohashi and Grotewold (2009); Pesch et al. (2015)]. As GL3 can homodimerize it is possible that also higher order complexes can be formed [Payne et al. (2000); Zhang et al. (2003); Bernhardt et al. (2003)]. In addition, the R3MYB TRIPTYCHON (TRY) and CAPRICE (CPC) proteins and others act as repressors of the MBW complex [Schellmann et al. (2002); Wang et al. (2008); Hülskamp et al. (1994); Wada et al. (1997); Zimmermann et al. (2004)].

The interaction between the MBW proteins and the differential formation of complexes is crucial for trichome patterning [Pesch et al. (2015); Dai et al. (2016); Struk et al. (2019); Brkljacic and Grotewold (2017); Fambrini and Pugliesi (2019); Wei et al. (2019); Morohashi and Grotewold (2009); Morohashi et al. (2007)]. The interactions between the WD40, MYB and bHLH proteins have been studied in different settings [Payne et al. (2000); Digiuni et al. (2008); Zhang et al. (2003); Zhao et al. (2008); Pesch et al. (2013)]. In yeast two-hybrid experiments, TTG1 and GL1 were both found to interact with GL3 but TTG1 and GL1 do not interact directly [Payne et al. (2000); Pesch et al. (2015)], leading to the hypothesis that GL1, GL3 and TTG1 form a trimeric complex together, capable of activating trichome differentiation events [Payne et al. (2000)]. It was later shown in yeast three-hybrid experiments and pulldown assays that GL1 and TTG1 counteract each other’s binding to GL3 [Pesch et al. (2015)], which led to a new model of differential dimer formation of GL3-GL1 and GL3-TTG1 dimers. Furthermore, these different dimers show a different extent of effect on the activity of the target promotors of TRY and CPC [Pesch et al. (2015)], indicating that the differential complex formation could be a mechanism of control, depending on the ratio of the dimers or higher order complexes. In addition, the R3MYB proteins compete with R2R3MYB for binding to GL3 [Pesch et al. (2015)]. Thus, there are two mechanistically different types of competition: the possible allosteric competition between WD40 and R2R3MYB and the competitive binding of the R2R3MYB and R3MYB proteins to GL3.

The current data indicate that the regulation of and by the MBW proteins is based on differential complex formation. As a consequence, the relative ratios of the MBW proteins should have a great impact on the formation of the MBW complexes and their regulation of target promoters [Morohashi et al. (2007); Morohashi and Grotewold (2009)]. This raises the question at which relative fractions the different protein complexes are formed. This would very much depend on the binding affinities of the protein-protein interactions. Quantitative binding studies using quartz crystal microbalance (QCM) revealed strikingly different dissociation constants between GL3 binding to GL1 and PAP1 [Umeyama and Morohashi (2020)]. Additionally, microscale thermophoresis experiments showed several fold differences in the binding affinity of GL3 and EGL3 to the inhibitor MYBL2 in the context of regulation of anthocyanin [Nemie-Feyissa et al. (2015); Zhou et al. (2019); Rajput et al. (2022)]. This prompted us to determine the relative dissociation constants of the proteins involved in trichome patterning by quantitative pull-down experiments. This enabled us to predict the relative percentage of all possible dimers and multimers at different ratios. In addition, we show by mathematical modelling that higher order complexes makes the patterning system more robust.

## 2 Methods

### 2.1 LUMIER (LUminescence-based Mammalian IntERactome)

*Staphylococcus aureus* protein A or *Renilla reniformis* luciferase (Rluc) was fused to the N-terminus of each protein while the third protein was fused to YFP at the N-terminus using the backbone of pTREXdest30 and three constructs were transiently expressed in HEK293TM cells (BioCat/SBI: LV900A-1). Transfection and cell harvesting were done as described before [Pesch et al. (2013); Pesch et al. (2015)]. After 48 hours cells were washed three times with PBS, lysed in 750*µl*-1000*µl* lysis buffer. Extracts were normalized with respect to the YFP signal and Rluc signal (TECAN) then combined after 1 hour lysis. The total volume was kept constant by adding untransfected cell lysate. Each combination was prepared in triplicate. Proteinimmunoprecipitation and luminescence measurements were done as described previously [Pesch et al. (2013)] using untransfected cells or cells expressing Luciferase-protein as controls. Cells solely expressing YFP-protein was also performed to exclude any nonspecific interference signal. The percentage of Rluc on the beads compared with the lysate was calculated by dividing the Rluc activity on the beads by the Rluc activity in the same amount of lysate used in the pull-down assay (input).

### 2.2 Western blot

Western blot experiments were performed as described in Molecular Cloning [Green and Sambrook (2012)]. Materials were used as follows: PVDF membrane (Roth), Super Signal West Femto Maximum Sensitivity Substrate (Termo Scientific), Mini Trans-Blot Cells (BioRad) for wet western blotting, Mini Protean Cells (BioRad) for SDS gel electrophoresis, and Prestained Protein Ladder (Fermentas). Protein lysate was extracted from HEK cell and detected with Anti-HA-Peroxidase (5 mU/ml 1:2500 roth). Each lane is 40*x* dilution of original lysate by lysis buffer. Relative density of each band is analyzed by ImageJ (1.48v, National Institutes of Health, USA).

### 2.3 Single site, reversible binding model

In the single binding experiments, the binding between the protA-tagged protein and the Renilla-tagged protein is measured. Since in every experiments we use the protA tag for GL3, we refer to it as GL3 henceforth. For fitting the data we use a simple, single-site binding model under the assumptions of mass balance and equilibrium. Let *x* stand for GL3, *y* for Renilla-tagged protein, *c* for the complex between *x* and *y*, *γ* for the association rate and *µ* for the dissociation rate, we get:


(1) $$x+y\overset{\gamma }{\underset{\mu }{⇌}}c$$

(2) $$\dot{x}=-\gamma xy+\mu c$$

(3) $$\dot{y}=-\gamma xy+\mu c$$

(4) $$\dot{c}=\gamma xy-\mu c$$

(5) $$x_{0}=x+c$$

(6) $$y_{0}=y+c,$$

where *x*_0_ *,y*_0_ is total amount of protein (the sum of free and bound protein). Upon substitution of Equations 5 and 6 into $\dot{x}=0$ we get the following expression for *c* at steady state:


(7) $$c=\frac{x_{0}}{2}\left[\left(1+\frac{y_{0}}{x_{0}}+\frac{\mu }{\gamma x_{0}}\right)-\sqrt{{\left(1+\frac{y_{0}}{x_{0}}+\frac{\mu }{\gamma x_{0}}\right)}^{2}-4\frac{y_{0}}{x_{0}}}\;\right].$$

Note that in Equation 7 the term $\frac{y_{0}}{x_{0}}$ indicates the ratio between the Renilla-tagged protein concentration *y*_0_ and GL3 concentration *x*_0_, and that the dissociation constant is given by $K_{D}=\frac{\mu }{\gamma }$ . So, given the normalization by the total amount of GL3 *x*_0_, we get a normalized *K_D_*, namely ${\bar{K}}_{D}=(\frac{\mu }{\gamma x_{0}})$ . Finally, to allow comparison between different experiments, we normalize *c* by *c_max_* which is the amount of complex at the point of saturation.

### 2.4 Competitive binding model

A protein binding experiment with competition for a single binding site is described by the following equations:


(8) $$x+y\overset{K_{Dy}}{⇌}xy$$

(9) $$x+z\overset{K_{Dz}}{⇌}xz,$$

where conservation of mass requires that


(10) $$x_{0}=x+xy+xz$$

(11) $$y_{0}=y+xy$$

(12) $$z_{0}=z+xz.$$

Assuming that the *x* protein is protA-tagged GL3 again and *y* is tagged with Renilla, we get the following expression used to fit to the data [Wang (1995)]:


(13) $$xy=\frac{{\bar{y}}_{0}\left[2\sqrt{a^{2}-3b}\cos(\theta /3)-a\right]}{3{\bar{K}}_{Dy}+\left[2\sqrt{a^{2}-3b}\cos(\theta /3)-a\right]},$$

where the bar notation indicates the normalization by *x*_0_ and


(14) $$a={\bar{K}}_{Dy}+{\bar{K}}_{Dz}+{\bar{y}}_{0}{\bar{z}}_{0}-1$$

(15) $$b={\bar{K}}_{Dz}\left({\bar{y}}_{0}-1\right)+{\bar{K}}_{Dy}\left({\bar{z}}_{0}-1\right)+{\bar{K}}_{Dy}{\bar{K}}_{Dz}$$

(16) $$c=-{\bar{K}}_{Dy}{\bar{K}}_{Dz}$$

(17) $$\theta =\text{arccos }\frac{-2a^{3}+9ab-27c}{2\sqrt{{\left(a^{2}-3b\right)}^{3}}}.$$

*K_Dy_, K_Dz_* are the same as the dissociation constants determined with the single binding model in Equation 7; in fitting the competition data, we allow these estimates to fall in the range given by the 95% confidence interval determined by the single binding model. Both the LUMIER data and the model Equation 13 are normalized by the amount of *xy* (i.e. the complex) measured at saturation levels.

### 2.5 Cooperative binding model

For the competition experiment with GL3, TTG1 and GL1 we first use the model in Equation 13 to try to fit the *K_D_* in the presence of the third protein. Because this does not give a good fit, the model is extended to include higher order complexes with the possibility of a GL1-GL3-TTG1 complex. Given that the *K_D_* for GL1 and TTG1 for binding to GL3 is very similar, we introduce a parameter *α* as a cooperativity parameter that indicates the change in the *K_D_* when GL1 or TTG1 is already bound to GL3, giving the following model:


(18) $$x+y\overset{K_{D1}}{⇌}xy$$

(19) $$x+z\overset{K_{D2}}{⇌}xz$$

(20) $$xz+y\overset{K_{D1/\alpha }}{⇌}xyz$$

(21) $$xy+z\overset{K_{D2/\alpha }}{⇌}xyz$$

(22) $$x_{0}=x+xy+xz+xyz$$

(23) $$y_{0}=y+xy+xyz$$

(24) $$z_{0}=z+xz+xyz.$$

Note that the binding of *y* to *x* is always indicated by *K_D_*_1_ as seen in Equations 18, 20, but in the case when *z* is already bound to *x*, the *K_D_*_1_ is adjusted by *α* as shown in Equation 20. Here the equations are solved numerically and *α* is estimated by a least squares fit. The interpretation of *α* can describe three different scenarios depending on its value: *1) α <* 1, the *K_D_* increases, i.e. negative cooperativity, *2) α* = 1 the *K_D_* is unchanged, i.e. no cooperativity but independent binding, *3) α >* 1, the *K_D_* decreases, i.e. positive cooperativity. In this case the signal is modelled as *xy* + *xyz* and is normalized by the signal at saturation. We fixed the range of the individual dissociation constants determined in the non-competitive experiments using the model in Equation 7 and the resulting 95% confidence interval, and estimate the cooperativity parameter *α* through non-linear least squares estimation.

### 2.6 Modelling higher order complexes in GL1

**Figure 1:**
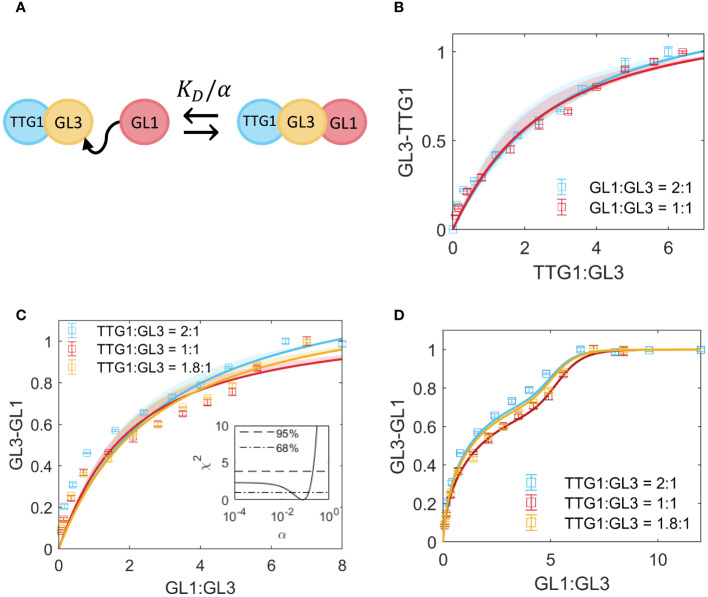
Competition between TTG1 and GL1 for binding to GL3. **(A)** Schematic representation of cooperativity between GL1 and TTG1. The *K_D_* of GL1 is adjusted by the cooperativity parameter *α* as a result of TTG1 bound to GL3. **(B)** Measurement of GL3-TTG1 binding in presence of GL1 at 2:1 (blue) and 1:1 (red) ratio. The model in A is used to fit the data and estimate *α* = 0.2. Shaded regions indicate the 95% confidence interval. **(C)** Measurement of GL3-GL1 binding in presence of TTG1 at 2:1 (blue), 1:1 (red) and 1.8:1 (yellow) ratio. The shaded region indicates 68% confidence interval as determined by the *χ* ^2^ profile of the estimate for *α* in the inset. The dashed line in the inset indicates the 95% confidence interval and the dash-dotted line indicates 68% confidence. **(D)** Fit to the data in C with a function that allows higher order complexes in GL1, given in Equation 25;

Given that the single site, reversible binding model showed a poor fit to the data in **Figure 1A**, we have used a model that allows homodimerization in GL1 such that higher order complexes than the trimer in **Figure 1C** can be formed. Towards this end, we introduce a hill-function [Ingalls (2013)] that describes binding in the following form:


(25) $$s=\frac{ax+bx^{n}+cxz+dx^{m}z}{1+ax+bx^{n}+cxz+dx^{m}z},$$

where *n* and *m* are the hill coefficients that indicate the order of protein *x* (in this case GL1), as a homodimer or as part of the complex with GL3 and TTG1, respectively; *a,b,c* and *d* are the coefficients for each binding term and *z* is the competitor protein (in this case TTG1). For **Figure 1B** the best fit is found for *n* = 1 and *m* = 10, indicating that a high form of non-linearity is part of the competitive complex formation which is more precisely defined in the following models.

### 2.7 Extension of the competitive displacement model

As the model in Equation 13 does not seem to reflect the experimental data of the competition between GL1 and the inhibitors very well, a different mechanism is explored. In this extension the binding between the inhibitor and GL1 is included. The set of reactions are:


(26) $$x+y\overset{K_{D1}}{⇌}xy$$

(27) $$x+z\overset{K_{D2}}{⇌}xz$$

(28) $$y+z\overset{K_{D3}}{⇌}yz$$

(29) $$x_{0}=x+xy+xz$$

(30) $$y_{0}=y+xy+yz$$

(31) $$z_{0}=z+xz+yz.$$

In this case it is not straightforward to derive an expression for the amount of complex like the one given in Equations 7, 13, therefore the equations are solved numerically to find the protein concentrations at steady state. Again the protein amounts and *K_D_* are normalized by *x*_0_, i.e. the total amount of protA-tagged GL3.

### 2.8 Extension of the cooperative binding model

To explore whether the homodimerization of GL3 plays an important role in the competition experiments with GL3, TTG1 and GL1 we extend the model from Equations 18-21 to include GL3 homodimerization. As a result of this extension the model now consists of 46 reversible reactions, where the highest order complex is a hexamer. We describe these reactions with a set of ordinary differential equations (ODEs) which, in chemical reaction network theory is commonly written as [Ingalls (2013)]:


(32) $$\dot{c}=S\vec{v}(c,k),$$

where S is the stoichiometric matrix and $\vec{v}\left(c,k\right)$ the vector of reaction rates, which are of the form of mass action kinetics:


(33) $$v_{j}=k_{j}\prod_{i=1}^{N}c_{i}^{\beta_{ij}},\;j=1,\ldots ,R,$$

where *N* is the number of species, *R* is the number of reactions and *β_ij_* is the molecularity of the reactant species *i* in reaction *j*. In this model we have *N* = 22 species and *R* = 92 reactions. Since we are dealing with a closed system at equilibrium we solve for $\dot{c}=Sv\left(c,k\right),$ to find the solutions of species concentrations at steady state. Cooperativity is only included when GL1 and TTG1 bind to the same sub-unit of GL3, for all other binding events we assume independent binding and use the *K_D_* estimates from the non-competitive data, including the GL3-GL3 binding rate. In the least squares fit to the data we have one estimable parameter, namely *α* the cooperativity parameter.

### 2.9 Parameter estimation and identifiability

In order to get an estimate for the *K_D_* the model output is fitted to the data via a least-squares approach [Kreutz et al. (2013)]. The agreement between data and model is described by the weighted sum of squared residuals:


(34) $$\chi^{2}\left(\theta \right)=\sum_{i=1}^{n}\frac{{\left(y_{i}-f\left(x_{i},\theta \right)\right)}^{2}}{\sigma_{i}},$$

where $y_{i}$ indicates the $i^{th}$ data point, $f\left(x_{i},\theta \right)$ the point as predicted by the model with parameters θ and $\sigma_{i}$ is the corresponding measurement error. The most optimal parameters $\hat{\theta }$ can then be estimated numerically by:


(35) $$\hat{\theta }=\text{arg min }\left[\chi^{2}\left(\theta \right)\right].$$

Confidence intervals for $\hat{\theta }$ can be derived by assuming a threshold in $\chi^{2}\left(\theta \right)$ , defined by the region [Kreutz et al. (2013)]


(36) $$\{\theta |\chi^{2}\left(\theta \right)-\chi^{2}\left(\hat{\theta }\right)<{\text{Δ}}_{\alpha }\}\text{ with }{\text{Δ}}_{\alpha }=\chi^{2}\left(\alpha ,df\right)$$

the threshold Δ*_α_* is the *α* quantile of the *χ* ^2^-distribution and with *df* the degrees of freedom, in this case *df* = 1, represents the confidence interval with confidence level *α*. This leads to a confidence interval for parameter *θ_i_* with lower bound $\sigma_{i}^{-}$ and upper bound $\sigma_{i}^{+}.\;\theta_{i}$ is identifiable if the interval $\left[\sigma_{i}^{-},\sigma_{i}^{+}\right]$ of its estimate ${\hat{\theta }}_{i}$ is finite [Kreutz et al. (2013)].

### 2.10 Multimers in the context of pattern formation: a mathematical analysis

We analyze the activator-inhibitor model as the typical reaction-diffusion scheme [Meinhardt and Gierer (1974); Turing (1952); Murray (2001)]:


(37) $$\partial_{t}u=f\left(u,v\right)+\nabla^{2}u=a-bu+\frac{u^{n}}{v}+\nabla^{2}u,$$

(38) $$\partial_{t}v=g\left(u,v\right)+d\nabla^{2}v=u^{n}-v+d\nabla^{2}v$$

where *a, b, n* and *d* are constants. This is a dimensionless version of the activator (*u*) - inhibitor (*v*) system. In the classical version of this system *n* = 2, but here we vary *n* to simulate higher-order complexes in the activator *u*. To determine what the effect is of increasingly higher order complexes on the pattern formation capabilities of the model, we derive the necessary conditions imposed on the model parameters by linear stability analysis [Turing (1952); Murray (2001)]. Instead of using any of the existing trichome models [Balkunde et al. (2020); Bouyer et al. (2008); Chopra et al. (2019); Digiuni et al. (2008); Pesch and Hülskamp (2009); Okamoto et al. (2020)], we use this simpler version as this allows an analytic approach in determining the conditions for the generation of spatial patterns. These conditions are [Murray (2001)]:


(39) $$f_{u}+g_{v}<0,\;f_{u}g_{v}-f_{v}g_{u}>0$$

(40) $$df_{u}+g_{v}>0,\;{\left(df_{u}+g_{v}\right)}^{2}-4d\left(f_{u}g_{v}-f_{v}g_{u}\right)>0,$$

where *f_u_, f_v_, g_u_* and *g_v_* are the partial derivatives of the reaction kinetics *f*(*u,v*) and *g*(*u,v*), evaluated at the uniform steady state. In the case of the activator-inhibitor system, this uniform steady state is


(41) $$u_{0}=\frac{a}{b},\;v_{0}={\left(\frac{a}{b}\right)}^{n}.$$

And at steady state the partial derivatives are


(42) $$f_{u}=\frac{b\left(n-a\right)}{a}$$

(43) $$f_{v}=\frac{-1}{{\left(\frac{a}{b}\right)}^{n}}$$

(44) $$g_{u}=n{\left(\frac{a}{b}\right)}^{n-1}$$

(45) $$g_{v}=-1.$$

Taken together, this leads to the following conditions:


(46) $$f_{u}+g_{v}<0\;\Rightarrow \;\frac{b\left(n-a\right)}{a}<1$$

(47) $$f_{u}g_{v}-f_{v}g_{u}>0\;\Rightarrow \;b>0$$

(48) $$\begin{array}{c} df_{u}+g_{v}>0\;\Rightarrow \;\frac{db\left(n-a\right)}{a}>1\\ {\left(df_{u}+g_{v}\right)}^{2}-4d\left(f_{u}g_{v}-f_{v}g_{u}\right)>0 \end{array}$$

(49) $$\Rightarrow \;{\left(\frac{db\left(n-a\right)}{a}-1\right)}^{2}>4db.$$

**Figure 2:**
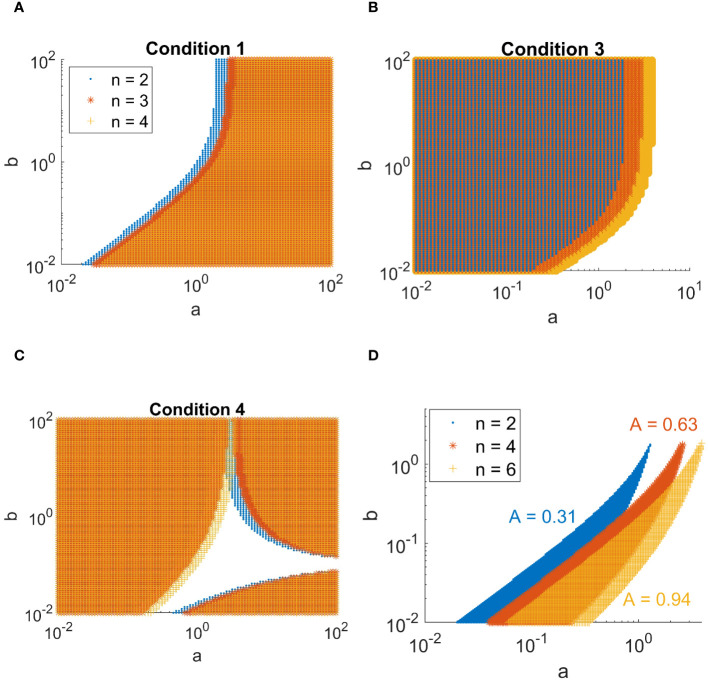
Combinations of *a* and *b* in the activator-inhibitor model in Equations 37 and 38 for which the patterning conditions are met. **(A-C)** Overview of the conditions derived in Equations 46-49. Values of *a* and *b* for which condition Equations 46, 48, and 49 are met are indicated by a marker. The different colors indicate the complex order *n* as shown in the legend of A. **(D)** Regions for which all conditions are met, for complex orders *n* = 2, 4, and 6. The size of the area *A* of each of the regions is indicated.

Upon fixing *d* = 10 and varying *a* and *b* across a wide range of values, the shape of the Turing space (the region in parameter-space where spatial patterns are generated) can be visualized as in **Figure 2D**, which is obtained by combining all the sub-conditions (**Figures 2A–C**) into one region. Note that the second condition, *b >* 0, will always be satisfied (a negative degradation rate *b* of the activator is not biologically relevant).

## 3 Results

### 3.1 Dissociation constants of TTG1-GL3 and GL1-GL3 dimers are in a similar range

**Figure 3:**
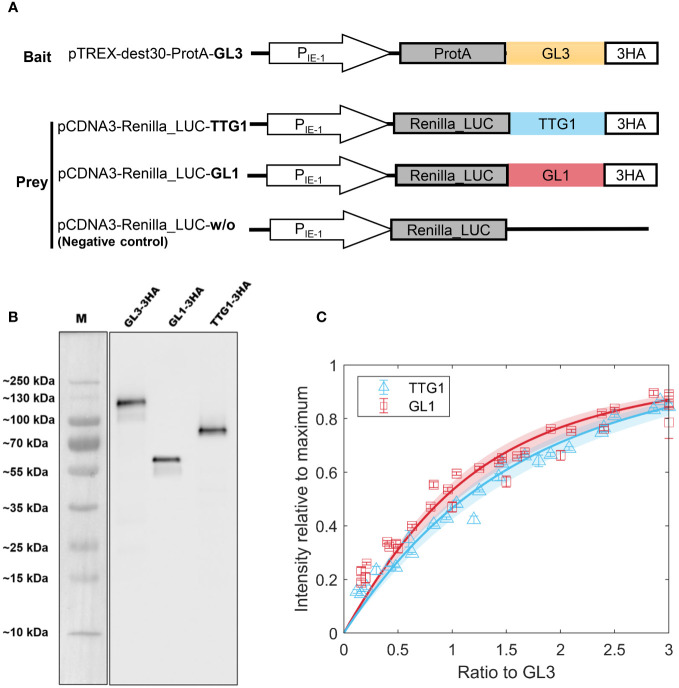
LUMIER binding assays to estimate dissociation constants. **(A)** Schematic representation of constructs used in LUMIER. **(B)** Western Blot used to determine the ratio of GL1 and TTG1 to GL1 using peak intensity. **(C)** Model fit to LUMIER data of GL1 (red) and TTG1 (blue) binding to GL3. The shaded region indicates the 95% confidence interval on the dissociation constant estimate. Error bars indicate 3 technical replicates. Cloud of points stems from three biological replicates which are not averaged into single points due to variation in expression levels.

To determine the relative amounts of the different complexes that can be formed by TTG1, GL3 and GL1, we aimed to determine the dissociation constants of the TTG1-GL3 and GL1-GL3 dimers. This requires a quantitative analysis of the protein concentrations at different ratios. As we have not been able to produce soluble TTG1, GL3 or GL1 proteins in sufficient amounts for a biochemical analysis, during the last years we developed an alternative approach using LUMIER pulldown assays (LUminescence-based Mammalian IntERactome [Blasche and Koegl (2013)]. This approach has proven to be very successful for the analysis of interactions between TTG1, GL3 and GL1 proteins [Pesch et al. (2015)]. ProtA-tagged GL3 and Renilla-tagged GL1 or TTG1 were expressed separately in human HEK293TM cells (**Figure 3A**), raw extracts were mixed and subjected to pulldown assays. Because we could not determine the absolute protein concentrations of GL1, GL3 and/or TTG1, we use a ratiometric approach. Towards this end, we added a HA-tag to all proteins and quantified the relative protein amounts of TTG1, GL3 and GL1 in parallel to the LUMIER assays on Western blots using the HA-antibody (**Figure 3B**, **Supplementary Figure 1**, **Supplementary Table 1**).

Based on these ratios we normalized the Renilla-tagged protein amounts with the GL3 levels. The ratio of GL1 or TTG1 to GL3 was varied by dilution series of the extracts. The amount of GL1 and TTG1 precipitated with ProtA-tagged GL3 was analyzed by measuring the Renilla luminescence. To enable a comparison between different experiments, we normalized every measurement by the maximum intensity. Each experiment included two technical replicas and was repeated several times (biological replicas), with different ranges of the ratios between GL1 or TTG1 and GL3. The data of the biological replicas were combined to estimate the dissociation constants (*K_D_*) for GL3-GL1 and GL3-TTG1. The K_d_ was calculated by fitting a model of reversible binding (Section 2.3, Equations 1–7) to the data with a non-linear least squares approach (**Figure 3C**). Because of the normalization methods that we used, these *K_D_* are dimensionless and relative to the total GL3 concentration. To indicate this, we refer to them as relative *K_D_*, denoted by ${\bar{K}}_{D}$ . The best fits resulted in a ${\bar{K}}_{D}=1$ for TTG1-GL3 and ${\bar{K}}_{D}$ = 0.5 for GL1-GL3. The TTG1-GL3 dimer has a slightly higher ${\bar{K}}_{D}$ than GL1-GL3. However, the confidence interval for both ${\bar{K}}_{D}$ estimates are very close. When taking into account that the method to determine the relative protein amounts by western blot analysis also introduces small errors we consider the dissociation constants of the protein dimers to be in a similar range.

### 3.2 Negative cooperativity between TTG1 and GL1 for binding to GL3

To quantify to what extent the binding of TTG1 to GL3 has an effect on subsequent binding of GL1 to GL3 and vice versa, we performed two assays. In the first approach, we added different amounts of TTG1-Renilla to GL3-ProtA in presence of GL1-YFP. As the Western blot analysis was done in parallel to the LUMIER assay, we could not calculate the GL1-YFP and GL3-ProtA ratios before starting the LUMIER analysis. The ratio of the GL1-YFP and GL3-ProtA varied between 1:1 to 1:2. First, we use the model in Section 2.4, Equations 8–17 to fit the competitive data. This model describes competition for a single binding site. Additionally, we test the model in Section 2.5, Equations 18–24, which includes the simultaneous binding of TTG1 and GL1 to a single GL3 unit (**Figure 1A**). Given that we find a better fit (according to the Akaike Information criterion, see **Supplementary Table 2**, which also includes the model fit for Equations 26–31 in Section 2.7), we assume this cooperativity model to be the most parsimonious for the competitive binding data. Fitting our data with the model revealed a cooperativity parameter of 0.2, indicating strong negative cooperativity (**Figure 1B**).

In the second approach, we quantified the GL1 GL3 interaction by adding different amounts of GL1Renilla to GL3-ProtA in the presence of TTG1-YFP. In all three experiments we found a slightly S-shaped response curve (**Figure 1C**), suggesting a highly non-linear behavior. Using the *χ* ^2^ score for the estimate of *α*, given in Equations 34–36, we found that the lower limit of the 95% confidence interval is unidentifiable [Kreutz et al. (2013)], as seen in the *χ* ^2^ profile for *α* (**Figure 1C**), which means that after a certain lower point in *α*, the fit of the model to the data is not improved upon further reduction of *α*. Given the poor fit of the model to the data, we asked the question what could result in the differing shape of the curve of the data and the model. One possible explanation is that GL1 shows weak homo-dimerization [Digiuni et al. (2008); Liang et al. (2014)] (**Supplementary Table 3**). When including the GL1 dimerization in our model (as described in Equation 25) we found the S-shaped response curve (**Figure 1D**).

### 3.3 Formation of higher order complexes of GL3

**Figure 4:**
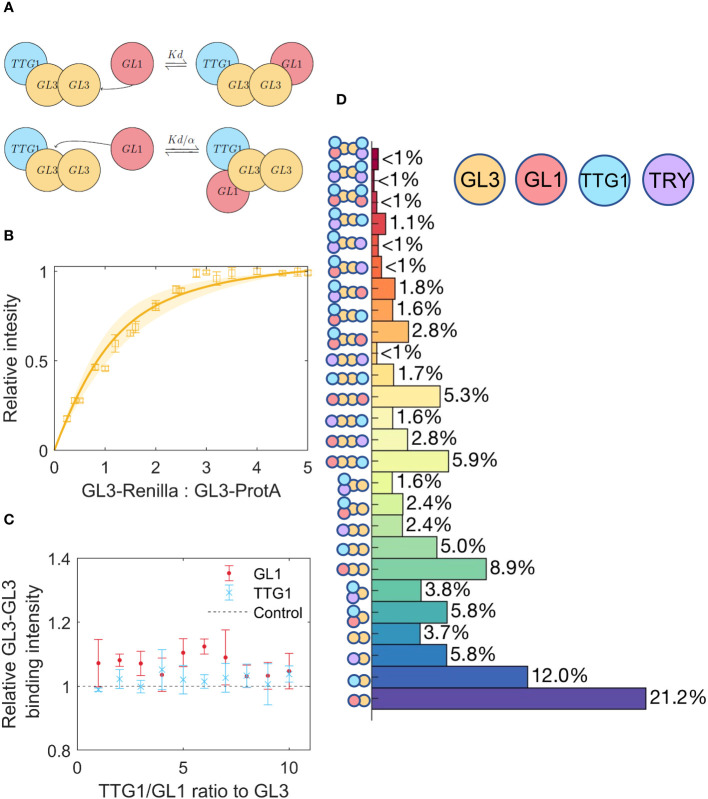
Competition between TTG1 and GL1 for binding to GL3, including GL3 homodimerization. **(A)** Schematic representation of cooperativity between GL1 and TTG1. The ${\bar{K}}_{D}$ of GL1 is adjusted by the cooperativity parameter *α* as a result of TTG1 bound to GL3. **(B)** Estimate of GL3-GL3 ${\bar{K}}_{D}$ using a least squares fit, the shaded region indicates the 95% confidence interval. **(C)** Measurement of GL3-GL3 binding under different amounts of TTG1 and GL1. Amount of GL3-GL3 binding is given relative to the control where TTG1 or GL1 is absent. **(D)** Prediction of the percentage of different possible complexes assuming equimolar amounts of GL1, TTG1, GL3 and TRY.

The homodimerization of GL3 could lead to higher order complexes [Bernhardt et al. (2003); Zhang et al. (2003); Zhao et al. (2008); Feller et al. (2011)], schematically represented in **Figure 4A**. In a first step we determine the ${\bar{K}}_{D}$ for the GL3 homodimer (**Figure 4B**). We find an estimate of ${\bar{K}}_{D}$ = 0.5, indicating that the GL3-GL3 dissociation constant is very similar to TTG1-GL3 and GL1-GL3.

In a next step we assessed whether GL3 dimerization is changed by additional binding of GL1 or TTG1. Towards this end, we performed LUMIER assays using GL3-ProtA and GL3-Renilla at different concentrations in the presence of TTG1-YFP or GL1-YFP (**Figure 4C**). These experiments revealed that GL1 and TTG1 do not change the dimerization behavior.

To enable the modelling of higher order complex formation mediated by GL3 dimerization, we limited the analysis on the competitive complex formation data obtained in the first set of TTG1-Renilla experiments. Note that for simplicity, we did not consider the potential higher order formation possibly mediated by the weak GL1 dimerization. The extended model takes into consideration competitive complex formation for the same GL3 protein, treats GL3 dimerization to be independent of binding to GL1 and/or TTG1 and assumes that GL1 or TTG1 binding to one of the GL3 molecules of the GL3 homodimer have no effect on binding sites of the other GL3 molecule (**Figure 4C**). The latter is plausible as GL3 dimerization is not affected by GL1 or TTG1. Modelling of these events revealed a cooperativity parameter of 0.4. Thus, GL3 dimerization resulted in a cooperativity parameter twice as high as calculated without GL3 homodimerization. This suggests that GL3 homodimerization reduces the predicted negative effect of GL1 on TTG1 binding and vice versa.

To determine which of the models (competitive or cooperative with or without GL3 homodimerization) explains the data most accurately we determined the root mean square error (RMSE) for each fit. Note that each of these models consists of the same amount of parameters to estimate. Although there was only a small difference, we found the lowest RMSE for the cooperative model with GL3 homodimerization (**Supplementary Table 4**).

### 3.4 Formation of the inhibitor complex

**Figure 5:**
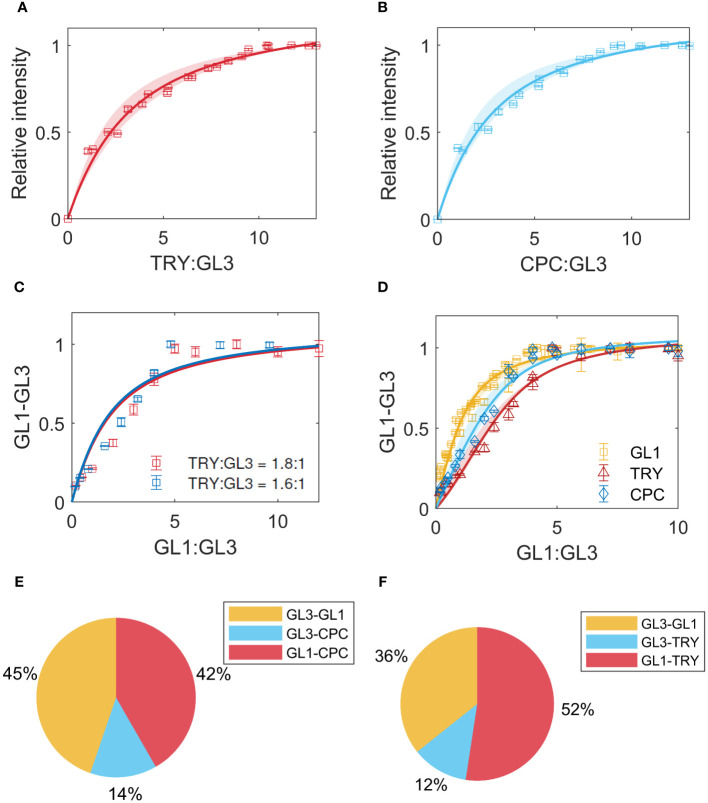
Inhibitor binding. **(A)** LUMIER data and model fit for TRY-GL3 binding. The shaded region indicates the confidence interval. **(B)** CPC-GL3 data and model fit. **(C)** Competition of GL1 with TRY, for different ratios indicated in legend. **(D)** Competition of GL1 with TRY (red) and CPC (blue), using a model that includes binding between the inhibitors and GL1. The yellow data indicates the GL1-GL3 binding in absence of TRY/CPC. **(E, F)** show the prediction of percentages of complexes formed using the estimates from the data for GL1 competition with TRY and CPC, respectively.

The binding of GL1 to GL3 is thought to occur at the same binding site as the inhibitors TRY and CPC [Zimmermann et al. (2004)]. To quantify this competition, we first determined the ${\bar{K}}_{D}$ of the two inhibitors with GL3 using GL3-ProtA in combination with TRY-Renilla or CPC-Renilla. For TRY and GL3 we found the best fit for ${\bar{K}}_{D}$ = 2.7 (**Figure 5A**), and for CPC-GL3 we found ${\bar{K}}_{D}$ = 2.3 (**Figure 5B**). As the confidence intervals overlap, we consider the ${\bar{K}}_{D}$ to be in the same range. Note, however, that the ${\bar{K}}_{D}$ of the inhibitors are approximately 2-fold higher than TTG1, GL1 and GL3 suggesting that the interaction between the activators is stronger than the interaction of the inhibitors with GL3.

In a next step we quantified the effect of the inhibitors on the binding between GL3 and GL1 experimentally. In these experiments, the binding between GL3-ProtA and GL1-Renilla is measured in the presence of a fixed amount of TRY-YFP or CPC-YFP. To find the best model to describe the data, we initially used the ${\bar{K}}_{D}$ values for GL1-GL3 and TRY/CPC-GL3 obtained in the non-competitive experiments in a competitive, single binding site model. This did not describe the data very well (**Figure 5C**). We therefore extended the model by testing whether the binding of the inhibitors to GL1 [Digiuni et al. (2008)] might improve the fit (**Figure 5D**), thereby introducing one parameter to estimate when fixing the estimates of binding to GL3 from the non-competitive data. This led to a clear reduction of the RMSE (**Supplementary Table 4**), indicating that the binding of the inhibitors to GL1 could be a potential explanation for the difference between the binding behavior predicted by the ${\bar{K}}_{D}$ obtained from pair wise interaction studies and three component assays. Finally, we use the ${\bar{K}}_{D}$ estimates to predict the percentages of the types of complexes formed when GL1, GL3 and TRY or CPC are present in equimolar amounts, including the binding between GL1 and TRY/CPC (**Figures 5E, F**).

### 3.5 Estimating the relative amounts of different MBW complexes

The complex interaction behavior of the MBW proteins and the inhibitors raised the question about the relative ratios of the different multimers and how these change with different ratios of the protein amounts. To estimate this, we used the ${\bar{K}}_{D}$ values to calculate the ratios of the multimers. In a first step, we calculated the relative amounts of MBW complexes without the inhibitor using the model described by Equations 32–33, Section 2.8. About 50% of the complexes would be expected to contain only one GL3 bound to GL1, TTG1 or both. Among the GL3 dimers containing complexes only a small fraction below 1% of all complexes are expected that contain six proteins (**Supplementary Figure 3**).

In a next step, we calculated the relative amounts of the multimers in the presence of TRY. When all proteins are present in equimolar amounts one would expect that 19% of the complexes with one GL3 protein contain TRY and are therefore expected to be inactive (**Figure 4D**). Among the GL3 dimer containing complexes about 23% of the complexes contain TRY suggesting that dimerization renders TRY inhibition slightly more effective.

Given that binding of TRY to GL3 is considered to represent the relevant biochemical mechanism of repression [Schellmann et al. (2002); Wang et al. (2008); Hülskamp et al. (1994); Wada et al. (1997)], it was surprising that only about 20% of the complexes contain TRY when all are present in equimolar amounts. We use a range of a relative changes between 0.25 to 4 of individual proteins with respect to GL3 (see **Supplementary App** to compare a wide range of combinations). This revealed some notable observations. First, the formation of GL1 GL3 TTG1 trimers is fairly low (about 5%) for a wide range of combinations and increases to more than 20% only, if the relative amounts of GL1 and TTG1 are both four-fold higher than GL3. Second, the amount of TRY containing inhibited complexes is between 15 and 30% for a wide range of concentrations. It requires low GL1 concentrations in combination with four-fold higher TRY levels to predict more than 50% inhibited complexes.

### 3.6 A patterning model predicts increased robustness through multimer formation

The largest complex included in this model consists of six sub-units. As all of these proteins are involved in the patterning of trichomes, a logical follow-up question is what the effect is of forming such a high order complex on the patterning capabilities of the trichome system. To make a prediction on this we study a simple model, namely the activator-inhibitor model [Meinhardt and Gierer (1974)]. This model has been used as a basic framework for trichome patterning to study competitive complex formation [Digiuni et al. (2008)], as well as other patterning-specific properties of the trichome network [Bouyer et al. (2008); Benitez et al. (2007); Benitez et al. (2008); Okamoto et al. (2020); Balkunde et al. (2020)]. At the basis of these models are the activator-inhibitor and substrate depletion systems [Meinhardt and Gierer (1974)]. Here, we have analyzed the activator-inhibitor model and adapted it to include the formation of higher-order complexes (*>* 2 sub-units) in the activator terms. We derived the conditions upon which the model would form patterns (see Methods, section 2.10, conditions derived in Equations 39–49), and determine how the size of the Turing space (in two dimensions) varies for complexes with *n* = 2, 4 or 6 sub-units. From this we find that a higher value for *n*, i.e. a higher order complex, leads to an increased size of the Turing space. This indicates that the number of possibilities of generating a pattern is increased. More specifically, patterning is more robust to changes in the values of *a* and *b* (production- and degradation-rate of the activator, respectively). Additionally, there is a shift in the patterning region towards higher values of *a*. This indicates that more activator is required to satisfy the conditions under high *n*.

## 4 Discussion

The regulation of trichome patterning is largely based on competitive complex formation such that the different trichome promoting complexes can be formed and these in turn can be repressed by competitive binding of inhibitor proteins [Pesch and Hülskamp (2009); Hülskamp (2004)]. Typically, this situation is sketched in a simplified manner: the three MBW proteins bind to the DNA to activate transcription and alternatively, the inhibitors replace the R2R3MYB to produce an inactive complex. This, however is unlikely to be the case in nature. One would rather expect that different amounts of the complexes are formed and that this depends on the relative amounts of the involved proteins and their binding affinities. To asses this, we determined the binding affinities and use them to calculate the relative fractions of all possible complexes. Towards this end, we used a quantitative LUMIER assay to determine the dissociation constants. Because GL3 is the platform for all binding partners [Payne et al. (2000); Zhang et al. (2003); Zimmermann et al. (2004); Feller et al. (2011); Morohashi et al. (2007); Morohashi and Grotewold (2009)] we could compare the dissociation constants as relative values, including the dimerization of GL3 and the binding of the inhibitors to GL3 [Schellmann et al. (2002); Wang et al. (2008); Hülskamp et al. (1994); Wada et al. (1997)]. By testing different modes of binding and competition, we determined the most likely scenarios given the data and predicted in what amount certain complexes are present when assuming a certain ratio of individual proteins.

### 4.1 Negative cooperativity between activators suggests higher order complex compositions

It is commonly assumed that a trimeric complex of GL3, TTG1 and GL1 drives the activation of downstream promoters that are involved in trichome patterning [Payne et al. (2000); Digiuni et al. (2008); Zhang et al. (2003); Zhao et al. (2008); Morohashi et al. (2007)]. From previously published results it was shown that there is a form of competition between GL1 and TTG1 in binding to GL3 [Pesch et al. (2015); Payne et al. (2000); Digiuni et al. (2008)] which led to the assumption that a differential dimer formation of GL3-GL1 and GL3-TTG1 is a more likely scenario. The results found in this paper support this hypothesis, as we found a negative cooperativity parameter between GL1 and TTG1.

Additionally, we found that the individual dissociation constants for both GL1 and TTG1 lie in similar ranges, indicating that their relative amounts play an important role in determining the final composition of complexes and thus the effciency with which certain downstream targets are activated [Dai et al. (2016); Struk et al. (2019); Brkljacic and Grotewold (2017); Fambrini and Pugliesi (2019); Wei et al. (2019)]. Finally, there is an added layer of complexity in the form of homodimerization of GL3 [Bernhardt et al. (2003); Zhang et al. (2003); Zhao et al. (2008); Feller et al. (2011)], which according to our binding models negates the effect of negative cooperativity between TTG1 and GL1, leading to higher-order complexes where both are found bound to either sub-unit of the homodimerized GL3. Taken together, the presented scenarios indicate a versatility in binding behaviors and a range of possible complexes in which the individual ratios of TTG1 and GL1 to GL3 play a crucial role in the final composition of complexes. This versatility could be translated into a fine-tuning mechanism where certain complex compositions lead to more or less efficient activation of targeted promoters. Furthermore, from a mathematical analysis using an activator-inhibitor patterning model we predict that the formation of higher-order complexes increases the robustness of the patterning system. The estimated increase of the size of the Turing space means that trichome patterning should still be possible when the system is challenged for example by changes in the expression strength of the key genes due to fluctuations or environmental stress. According to the eFP Browser [Winter et al. (2007)] all trichome patterning genes are up- or down-regulated in one or more stress conditions. The predicted increase in robustness due to the higher-order complexes may represent one aspect to safeguard trichome formation under different life conditions. Note that the observations made for this simple activator-inhibitor system do not necessarily extend to the models formulated for the trichome system. This analysis can only indicate a tendency in the changes in patterning space as a result of higher order complex formation. Its strengths lie in its simplicity and thus the ease of interpretation. For any of the more extensive and intricate trichome models, such an analysis would become intractable. However, as the activator-inhibitor system underlies the trichome patterning models [Bouyer et al. (2008); Digiuni et al. (2008); Pesch and Hülskamp (2009); Hülskamp (2004); Benitez et al. (2008); Balkunde et al. (2020)], it is conceivable to use it as an appropriate simplification.

### 4.2 Inhibitors binding is weaker than activators and show a complexity beyond competitive binding

The estimated dissociation constants of TRY and CPC are 2-fold higher than found for GL1 and TTG1, indicating weaker binding to GL3. As the displacement of GL1 by TRY and CPC is expected to be the leading mechanism by which TRY and CPC exert their inhibitory function [Digiuni et al. (2008); Zimmermann et al. (2004); Schellmann et al. (2002); Wang et al. (2008)], this would suggest that in order to achieve inhibition in non-trichome cells, the inhibitors would have to be present in higher amounts than the activators in order to compensate for the weaker binding strength. This is in line with the concentration profiles predicted by the patterning models based on activator-inhibitor principles assumed for trichome patterning, where in the trichome-peak the amount of activators is much higher than inhibitors and vice versa in epidermal cells [Digiuni et al. (2008); Bouyer et al. (2008); Benitez et al. (2007); Okamoto et al. (2020); Balkunde et al. (2020)]. While this difference in binding strength could play a role in achieving this difference, it is not the only mechanism in the patterning models. More specifically, these models include feedback-loops to achieve this distribution of high activator in trichome cells and high inhibitor in non-trichome cells [Digiuni et al. (2008); Bouyer et al. (2008); Benitez et al. (2007); Okamoto et al. (2020); Balkunde et al. (2020)].

### 4.3 New questions

We distinguish 26 possible complexes and predict that they occur with fractions between about 1% and 21% when all proteins are considered to be present in equimolar ratios. Even when changing the relative contributions drastically, the relative fractions of some complexes increase, however, fairly moderately. This raises several questions of which we like to highlight three: First, what are the ratios of the MBW proteins and their inhibitors during the patterning process? Second, which of the complexes is biologically active? Third, how can these data be integrated in more complex models to consider the complex formation in a quantitative manner. All three questions are experimentally very challenging, however, appear to be essential for the mechanistic understanding of the process.

## Data availability statement

The original contributions presented in the study are included in the article/**Supplementary Material**. Further inquiries can be directed to the corresponding authors.

## Author contributions

BZ: Conceptualization, Formal analysis, Investigation, Writing – original draft. AD: Conceptualization, Data curation, Formal analysis, Investigation, Methodology, Software, Visualization, Writing – original draft. CF: Conceptualization, Funding acquisition, Project administration, Supervision, Writing – review & editing. MH: Conceptualization, Funding acquisition, Project administration, Resources, Supervision, Writing – review & editing.

## Supplementary material

Supplementary App A GUI written in Python in which the results are visualized is available at https://github.com/MathModelTrichome/MBW-Complex-Formation.
