# Supplementary material for: Quantitative analysis of MBW complex formation in the context of trichome patterning
Source: Front Plant Sci. 2024 Mar 5;15:1331156. doi: 10.3389/fpls.2024.1331156 (PMC10948613; doi:10.3389/fpls.2024.1331156)
Supplement: Supplementary App — A GUI written in Python in which the results are visualized is available at https://github.com/MathModelTrichome/MBW-Complex-Formation. [file DataSheet_1.pdf]

Supplementary Material

1 SUPPLEMENTARY INFORMATION

Model selection

To determine which of the models explains the TTG1, GL3, GL1 competition most accurately we use the Akaike Information Criterion (AIC) (Akaike (1998)) as a measure for model parsimony. The AIC is given by

$$\mathrm{AIC} = 2P - 2\ln(\mathcal{L}), \tag{S1}$$

with P the number of parameters in the model and $\ln(\mathcal{L})$ the log-likelihood of the model fit to the data. The negative log-likelihood is given by

$$-\ln(\mathcal{L}) = \frac{N}{2}\ln(2\pi) + \frac{N}{2} + \frac{N}{2}\ln\left(\frac{\mathrm{RSS}}{N}\right), \tag{S2}$$

where RSS is the residual sum of squares from the least-squares fit and N the number of data points (Portet (2020)). For the data with GL3-ProtA, TTG1-Renilla and different amounts of GL1-YFP (Figure 2B), we compare both the competitive model and the cooperative model and select the most parsimonious model based on the lowest AIC value (Supplementary Table S2). Note that since we use the same data for the two models, we can simplify Eq. (S1) to

$$\mathrm{AIC} = N\ln\left(\frac{\mathrm{RSS}}{N}\right) + 2P. \tag{S3}$$

2 SUPPLEMENTARY FIGURE 1

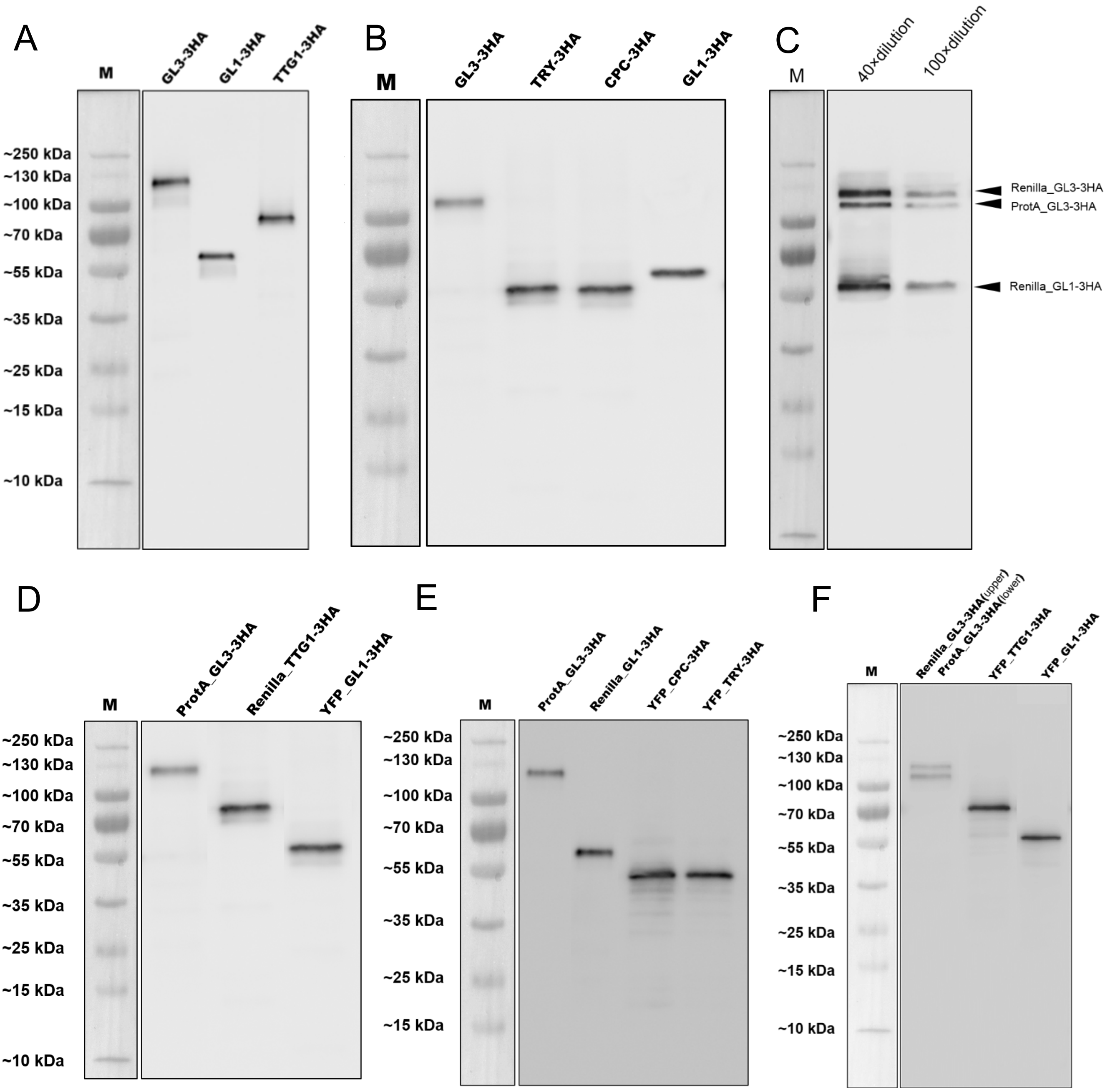

Figure S1. Western blot analysis of proteins expressed in HEK cells. Protein lysate was extracted from HEK cells and detected with Anti-HA-Peroxidase (5 mU/mL 1:2500 roth). Each lane is a 40x dilution of the original lysate by lysis buffer. Relative density of each band is analysed by ImageJ (v1.48, National Institute of Health, USA), see Supplementary Table 1.

3 SUPPLEMENTARY FIGURE 2

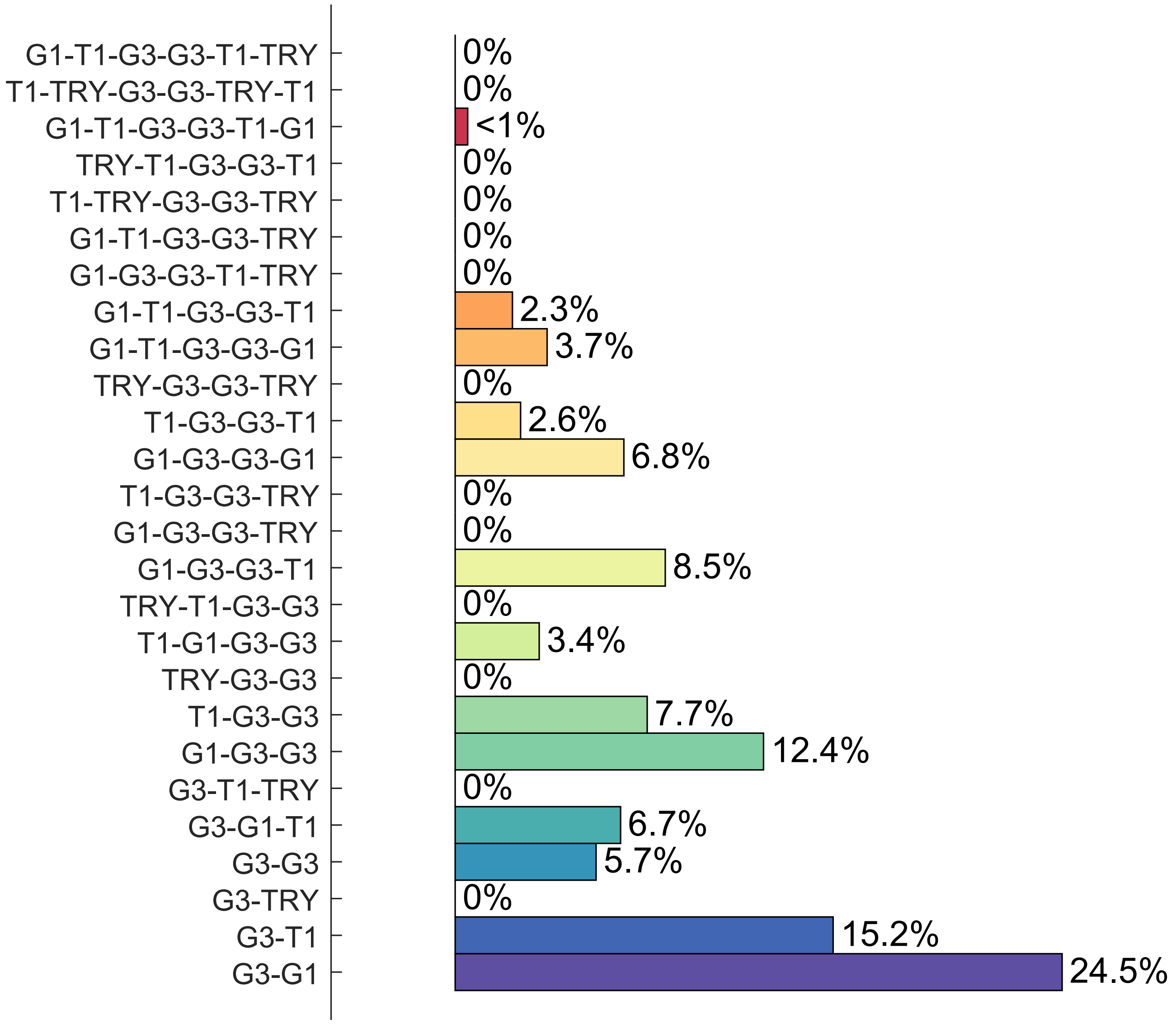

Figure S2. Prediction of percentages of complexes found in absence of inhibitors. Percentages of complexes found are predicted using estimates of dissociation constants determined from LUMIER experiments. As ratio of components we assumed GL3:GL1:TTG1:TRY as 1:1:1:0, i.e. the activators are present in equimolar amounts and there is no inhibitor.

4 SUPPLEMENTARY TABLE 1

Table S1. Band intensities of Western blots in Figure S1

Sample	Peak intensity [%]	Relative density
**Figure S1 A**		
ProtA_GL3-3HA	18.612	1.000
Renilla_GL1-3HA	15.394	0.827
Renilla_TTG1-3HA	15.541	0.835
**Figure S1 B**		
ProtA_GL3-3HA	10.752	1.000
Renilla_TRY-3HA	16.342	1.520
Renilla_CPC-3HA	16.456	1.531
Renilla_GL1-3HA	17.003	1.581
**Figure S1 C**		
ProtA_GL3-3HA	7.121	1.000
Renilla_GL3-3HA	8.462	1.188
Renilla_GL1-3HA	8.771	1.232
**Figure S1 D**		
ProtA_GL3-3HA	10.106	1.000
Renilla_TTG1-3HA	18.132	1.794
Renilla_GL1-3HA	13.993	1.385
**Figure S1 E**		
ProtA_GL3-3HA	8.996	1.000
Renilla_GL1-3HA	13.167	1.464
YFP-CPC-3HA	14.722	1.637
YFP-TRY-3HA	14.672	1.631
**Figure S1 F**		
ProtA_GL3-3HA	4.111	1.000
Renilla_GL3-3HA	4.098	0.997
Renilla_TTG1-3HA	14.593	3.550
Renilla_GL1-3HA	14.281	3.474

5 SUPPLEMENTARY TABLE 2

Table S2. Akaike Information Criterion (AIC) for the competitive and cooperative model for two datasets. N indicates the number of data points, P the number of parameters and RSS the residual sum of squares.

Model	N	P	RSS	AIC
Competitive binding	26	2	0.0688	-150
Cooperative binding	26	3	0.0594	-152

6 SUPPLEMENTARY TABLE 2

Table S3. LUMIER experiment of GL1 homodimerization

ProtA_GL1	Renilla_GL1	Input (mean)	Pull down	Interaction
50 uL	50 uL	1396	268	-
50 uL	100 uL	2107	403	slightly
50 uL	200 uL	4022	695	+

7 SUPPLEMENTARY TABLE 4

Table S4. Root mean square error (RMSE) of different models and datasets.

Model	Dataset	RMSE
Cooperativity, trimer	TTG1-GL3 measured, GL1 fixed	0.0588
Cooperativity, hexamer	TTG1-GL3 measured, GL1 fixed	0.0476
Competition with inhibitor	GL1-GL3 measured, TRY fixed	0.1025
Competition with inhibitor	GL1-GL3 measured, CPC fixed	0.0761
Competition with inhibitor, GL1-Inhibitor binding	GL1-GL3 measured, TRY fixed	0.0584
Competition with inhibitor, GL1-Inhibitor binding	GL1-GL3 measured, CPC fixed	0.0545

REFERENCES

Akaike, H. (1998). Information theory and an extension of the maximum likelihood principle. In *Selected papers of hirotugu akaike* (Springer). 199–213

Portet, S. (2020). A primer on model selection using the akaike information criterion. *Infectious Disease Modelling* 5, 111–128
